# Anatomical Relationships of the Proximal Attachment of the Hamstring Muscles with Neighboring Structures: From Ultrasound, Anatomical and Histological Findings to Clinical Implications

**DOI:** 10.3390/diagnostics14161725

**Published:** 2024-08-08

**Authors:** Maribel Miguel-Pérez, Pere Iglesias-Chamorro, Sara Ortiz-Miguel, Juan-Carlos Ortiz-Sagristà, Ingrid Möller, Joan Blasi, Josep Agullò, Carlo Martinoli, Albert Pérez-Bellmunt

**Affiliations:** 1 Unit of Human Anatomy and Embryology, Department of Pathology and Experimental Therapeutics, Faculty of Medicine and Health Sciences (Bellvitge Campus), Universitat de Barcelona, 08907 Barcelona, Spain; centretars@centretars.com (P.I.-C.); ingridmoller@ub.edu (I.M.); jlagullo-1@ub.edu (J.A.); 2 Basic Sciences Department, Universitat Internacional de Catalunya, 08017 Barcelona, Spain; sara.mortiz@ub.edu (S.O.-M.); aperez@uic.cat (A.P.-B.); 3 ACTIUM Functional Anatomy Group, Sant Cugat del Vallés, 08195 Barcelona, Spain; 4 Unit of Human Anatomy and Embryology, Department of Surgery and Medical-Surgical Specialities, Faculty of Medicine and Health Sciences (Clinic Campus), University of Barcelona, 08036 Barcelona, Spain; 5 Euses, Bellvitge Campus, Universitat de Barcelona, 08907 Barcelona, Spain; 6 Anesthesia Department, Fundació Puigvert, 08025 Barcelona, Spain; siometge@gmail.com; 7 Unit of Histology, Department of Pathology and Experimental Therapeutics, Faculty of Medicine and Health Sciences (Bellvitge Campus), Universitat de Barcelona, 08907 Barcelona, Spain; blasi@ub.edu; 8 Dipartimento di Scienze della Salute, Universita di Genova, 16126 Genoa, Italy; carlo.martinoli@unige.it

**Keywords:** hamstring, adductor magnus, ischial tuberosity, sacrotuberous ligament, biceps femoris, musculoskeletal, tendinopathy, sciatic nerve, ultrasound

## Abstract

Background: Injuries of the proximal attachment of the hamstring muscles are common. The present study aimed to investigate the relationship of the proximal attachment of the hamstring muscles with neighboring structures comprehensively. Methods: A total of 97 hemipelvis from 66 cryopreserved specimens were evaluated via ultrasound, anatomical and histological samples. Results: The proximal attachment of the hamstring muscles presents a hyperechogenic line surrounding the origin of the semimembranosus and the long head of the biceps femoris muscles, as well as another hyperechogenic line covering the sciatic nerve. The anatomical and histological study confirms the ultrasound results and shows different layers forming the sacrotuberous ligament. Furthermore, it shows that the proximal attachment of the semimembranosus muscle has a more proximal origin than the rest of the hamstring muscles. Moreover, this muscle shares fibers with the long head of the biceps femoris muscle and expands to the adductor magnus muscle. The histological analysis also shows the dense connective tissue of the retinaculum covering the long head of the biceps femoris and semimembranosus muscles, as well as the expansion covering the sciatic nerve. Conclusions: These anatomical relationships could explain injuries at the origin of the hamstring muscles.

## 1. Introduction

The ischial tuberosity is a sizeable round protrusion at the inferoposterior aspect of the ischium of the coxal bone and is an origin of powerful muscle groups [1]. The semimembranosus (SMB), the long head of the biceps femoris (LHBF) and the semitendinosus (ST) muscles, forming the hamstrings muscles, have their proximal attachment in this zone [2], which is the most frequently injured area of these muscles [3,4,5,6].

For clinical implications, the anatomy of this zone has been re-examined and reanalyzed recently by different studies. Some studies have described an annular structure surrounding the hamstring muscles [7], or the presence of an anatomical and histological continuity between the LHBF and the sacrotuberous ligament (STL) [8] with similar ratios of collagen and elastic fibers between this ligament and the LHBF [9]. The morphometry, morphology and relationships of the proximal attachment of the hamstring muscles have also been analyzed [10,11,12,13]. However, these recent investigations still have not described possible anatomical relationships that could explain some specific pathologies or pathological associations.

An essential aspect of clinical diagnosis following an injury is to visualize the muscles through radiological methods. It is the gold standard to confirm their normality and check the severity and prognosis of the hamstring injury [14]. One of the most widely used techniques for imaging the proximal hamstrings is ultrasound scanning [15,16,17]. However, despite the importance of these muscles and their new connections described in recent studies, there are no studies that have used ultrasound to describe the new anatomical findings to the best of our knowledge.

The current study aimed to broaden the ultrasound and anatomical knowledge of the attachment of different structures in the ischial tuberosity, focusing on the proximal attachment of the hamstring muscles and its relationships with other anatomical structures. This information may help the exploration, clinical assessment and treatment of the different anatomical structures with anchorage at this point.

## 2. Materials and Methods

This study was performed on specimens from 97 hips (37 females and 60 males) from 66 adult humans with a mean age of 78.6 years at death. The specimens were cryopreserved at −20 °C in the dissection room before we studied them at ambient temperature. All 97 ischial tuberosities and proximal attachments of the hamstring muscles were analyzed. There was no evidence of deformities, traumatic injury or surgical scarring in any of the specimens studied. The body donor signed a document to participate in the body donation program of the Faculty of Medicine and Health Sciences. Appropriate consent was obtained and approved by the local ethics committee. This study was conducted in accordance with the principles of the Declaration of Helsinki.

This study consisted of three stages: the first stage was an ultrasound study of the origin of hamstrings muscles at the ischial tuberosity focusing on the semimembranosus and long head of the biceps femoris muscles and their relationship with other anatomical structures as the adductor magnus muscle and sciatic nerve; the second stage was the anatomical study; and the third stage was a histological study of the retinaculum and expansion over the sciatic nerve.

### 2.1. Ultrasound Study

The ultrasound study was performed with a General Electric LOGIQ P6 and P9 ultrasound scanner (GE Ultrasound Korea. Ltd., Seongnam, Republic of Korea) with a high-frequency 6–15 MHz linear probe that was lower in the obese specimens. The specimens were lying prone, with their feet hanging off their stretched legs. The posterior axial planes are the most useful for visualizing the ischial tuberosity and the insertion of the proximal origin of the tendons of the hamstring muscles on its lateral aspect, as recommended by the European Society of Musculoskeletal Radiology [18]. After locating the ischiatic tuberosity with the probe in the short axis, the origin of the hamstring muscles was located. With the probe in this position, the medial and lateral facet of the ischial tuberosity was visualized. In this position, the LHBF and SMB were identified, although the probe was moved 30–45° clockwise on the right side and on the left side in some specimens to try to identify the classical image described by the European Society of Musculoskeletal Radiology after the STL and the origin of the SMB and LHBB had been identified. Guided by ultrasound and parallel to the probe, 3 mL of dye (1 mL of a red or green dye with 2 mL of saline) was injected with a 20 G needle at the SMB and LHBF. Also, the superior insertion of the SMB was located with the probe in the long axis, and 3 mL of the dye was injected at his point.

The relationship between the sciatic nerve and the hamstring origin was studied in relation to the retinaculum above the hamstring origin. At this last lateral point, 3 mL of the red dye was injected, guided by ultrasound.

The thicknesses of the retinaculum and expansions covering the sciatic nerve were measured.

### 2.2. Anatomical Procedure

The anatomical study was carried out using two different techniques. A total of 85 hemipelves were analyzed via anatomical dissection, while 11 was analyzed using sectional anatomy. At each step, photographs were taken to record information (Canon EOS 60D, Kyanon Kabushiki-kaisha. Ota, Tokyo, Japan).

#### 2.2.1. Dissection Procedure

The dissection of the proximal attachment of the hamstring muscles was performed stratigraphically in all the specimens, with special care taken to locate and prove that the dye was in the correct tendon. The anatomical dissection was performed following the classic method in planes with a longitudinal medial incision made in the middle posterior line of the body and two horizontal incisions made at the iliac crest and in the middle of the posterior side of the thigh. The gluteus maximus was then removed from the medial origin to the lateral insertion on the femur. The correct localization of the dye, the relationship between the proximal attachment of these muscles and the ischial tuberosity and the relationships between them and their surrounding connective and fascial tissues were meticulously examined, especially the connections with the STL and the sciatic nerve.

#### 2.2.2. Anatomical Sections

Ten hemipelves from five specimens were used to make transversal anatomical cross-sections at the level of the ischial tuberosity.

### 2.3. Histological Study

In 7 randomly chosen specimens, we collected samples of comparable size (2 cm × 2 cm) from the STL in association with the LHBB, the retinaculum over LHBF and SMB and the expansions covering the sciatic nerve. The samples were fixed in 4% formaldehyde, processed into paraffin blocks and cut into 4 µm sections before being dyed with a hematoxylin-eosin stain. The thicknesses of the retinaculum and expansions were measured with a Leica digital microimaging device (Leica DMD108 microscope, Leica Microsystems GmbH, Wetzlar, Germany).

The thicknesses of the retinaculum and expansions covering the sciatic nerve were measured.

### 2.4. Statistical Analysis

Statistical analysis of the thicknesses of the retinaculum and the expansion of the sciatic nerve was performed in 43 specimens (17 females and 26 males; 20 right and 23 left side) on all the data obtained for the control variables (i.e., sex, limb side and age) and the anthropometric variables.

The qualitative variables were presented as absolute and relative frequency; quantitative variables were presented as the median and interquartile range (IQR) and the mean with standard deviation. The Shapiro–Wilk test was used to study the existence of normal distribution in quantitative variables. The relationship between categorical and quantitative variables is carried out through Student’s T-test (normal distribution) or Mann–Whitney U test (non-normal).

## 3. Results

In total, 97 ischial tuberosities and proximal attachments of the hamstring muscles from 66 cryopreserved specimens (37 females and 60 males; 53 right and 44 left sides) with a mean age of 78.6 (range 48–89 years) were analyzed. Measurements were recorded in 43 specimens. Among these, 26 were from men (60.5%) and 17 were from women (39.5%), while 20 were right-sided (46.5%) and 23 were left-sided (53.5%). The thickness measurements of the retinaculum and expansion over the sciatic nerve via ultrasound were 0.57 mm and 0.20 mm, respectively (Table 1). Statistical analysis showed that neither gender nor laterality affected these measures. (Table 2 and Table 3).

**Figure 1:**
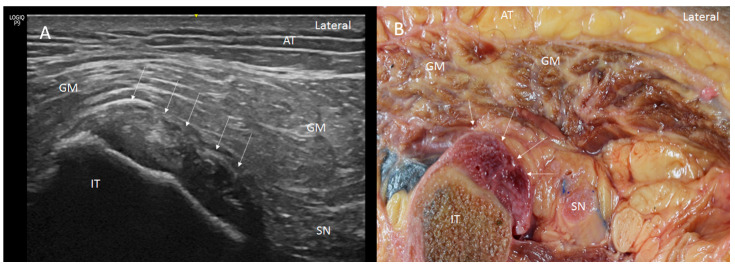
(**A**) Ultrasound view in short axis of the right tendon of long head of biceps femoris (LHBF) and semimembranosus (SMB) muscles seen as two round hyperechogenic structures similar to a mask or the shape of a goat’s leg at the ischial tuberosity (IT). They are surrounded and isolated by a hyperechogenic line that fixed them to the bone (white arrows). Adipose tissue (AT), gluteus maximus muscle (GM), sciatic nerve (SN), (**B**) Anatomical section of the hip showing the LHBF and SMB tendons fixed by the retinaculum (white arrows).

**Figure 2:**
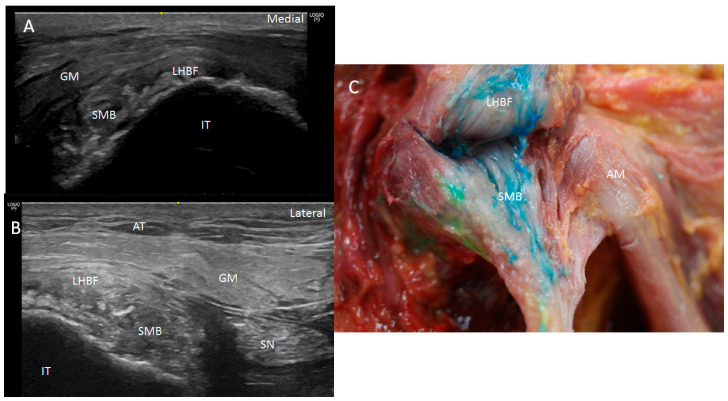
(**A**,**B**) Two ultrasound examples in the short axis of the tendons of the long head of biceps femoris (LHBF) and the semimembranosus (SMB) muscles, presenting an irregular origin at the ischial tuberosity (IT) with several hyperechogenic structures that were compatible with a degeneration of the tendon: (**A**) left side; (**B**) right side. (**C**) Dissection of the left area showing, after moving the LHBF, a very irregular insertion of the tendon of the SMB with all the blue dye widespread. AM: adductor maximus muscle.

**Figure 3:**
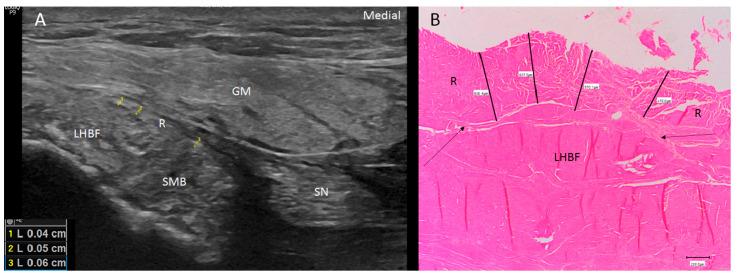
(**A**) Ultrasound view of the retinaculum (R) as a hyperechogenic line that fixed them to the bone (as well as the different points where it was measured (yellow numbers 1, 2 and 3). Tendon of long head of biceps femoris (LHBF) and semimembranosus muscle (SMB). Right side: gluteus maximus muscle (GM). (**B**) Histological view of the retinaculum and its measures of 636.4 µ 637.9 µ, 570.1 µ and 470 µ. It is composed of dense connective tissue, and it is possible to observe the perpendicular direction of its fibers with the fibers of the tendon of LHBF and their close relation (black arrows).

Ultrasound showed two hyperechogenic lines, lateral and media, corresponding to the ischial tuberosity. In the lateral line, the proximal origin of the SMB and LHBF were observed as two round hyperechogenic structures (contiguous and adjacent to one another), similar to a mask (Figure 1). The superomedial structure corresponded to the LHBF tendon, while the inferolateral structure corresponded to the SMB tendon (Figure 1). However, in some cases (30 from 97 hemipelvis (32.3%)), this image was not clear, and the probe had to be turned 30° with respect to the short axis (anticlockwise on the right side and clockwise on the left side). In 25.5% (38 from 97 cases) of the specimens, the SMB, and even the LHBF, did not show a well-defined structure and presented several hyperechogenic structures that were compatible with a degeneration of the tendon (Figure 2). In these specimens, the dye had spread more. In all the specimens, both tendons (SMB and LHBF) were surrounded and isolated by a hyperechogenic line that fixed them to the bone (Figure 3). The thickness of this line was 0.57 mm (Table 1).

**Figure 4:**
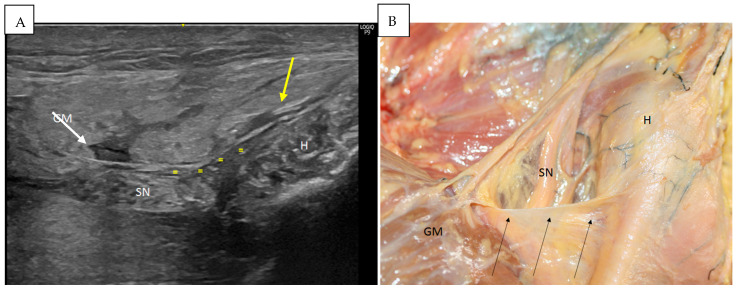
(**A**) The expansion over the sciatic nerve (SN) on the left side. An ultrasound study allows us to visualize this expansion (yellow =), which goes from the origin of the hamstrings (H) (yellow arrow) to the gluteus maximus muscle (GM) (white arrow). IT: Ischial tuberosity. (**B**) Anatomical study allowing us to see this expansion (black arrows) over the sciatic nerve on the left side. It is possible to visualize that it goes from the origin of the hamstrings (H) (yellow arrow) to the gluteus maximus muscle (GM) (white arrow).

From this point, when the probe was moved laterally in the short axis, it was possible to visualize a very thin hyperechogenic line ranging from the ischial tuberosity and the origin of the hamstrings to the gluteus maximus muscle that covered the passageway of the sciatic nerve and the posterior femoral cutaneous nerve (Figure 4). The thickness of the expansion was 0.2 mm (Table 1).

When the ultrasound study of the specimen was followed inferiorly, we observed that the LHBF was always more superficial and posterior, and the SMB tendon turned to the medial position. SMB went to a deep and medial position in the anterior face of the ST origin. At this point, we observed the muscular fibers of the ST occurring medially to the LHBF and covering the SMB tendon.

**Figure 5:**
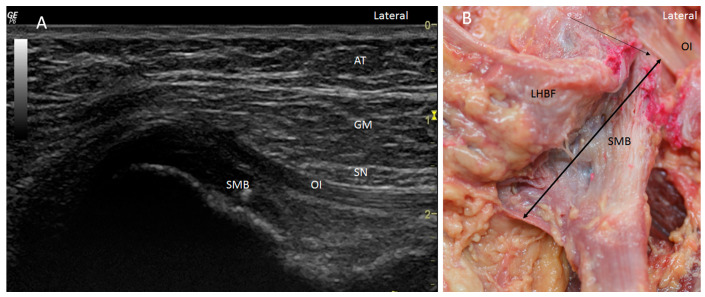
(**A**) Ultrasound study in the short axis on the right side showing the high origin semimembranosus muscle (SMB). It goes up even as far as to be placed under the obturator internus muscle (OI). SN: sciatic nerve GM: gluteus maximus muscle; AT: adipose tissue. (**B**) Anatomical view of the long and high origin of the tendon of the SMB (black arrow) under the OI after moving the LHBF medially on the right side.

**Figure 6:**
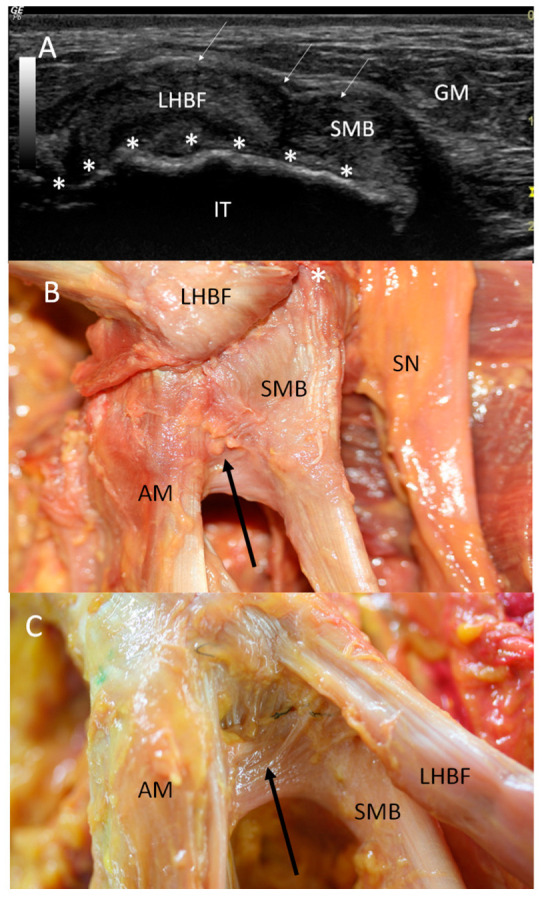
(**A**) Ultrasound view in the short axis showing the high origin of the right tendon of the semimembranosus muscle (SMB) (white *) at the ischial tuberosity (IT). LHBF: the tendon of the long head of biceps femoris; white arrow: retinaculum (white arrow); GM: gluteus maximus muscle. (**B**) Anatomic view of the origin after moving up the LHBF; it is possible to visualize the high origin of the SMB (white *) and the deep connections (black arrow) with the origin of the adductor maximus muscle (AM) (laterally, the sciatic nerve). (**C**) Anatomic view to observe the deep relation between LHBF and the SMB and the SMB and AM (black arrow). Both anatomical views are on the right side.

When the probe followed the SMB superiorly in the short axis, we observed its long and superior insertion, showing a deep relationship with the LHBF, quadratus and obturator internus muscles (Figure 5 and Figure 6).

**Figure 7:**
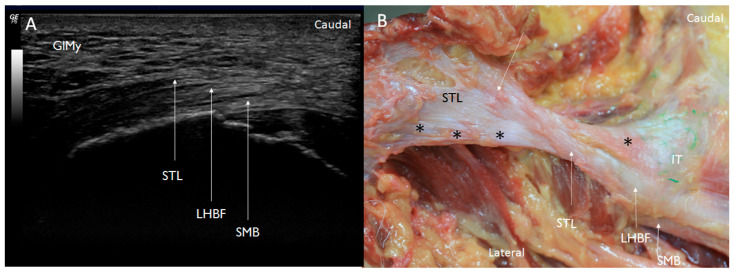
(**A**) Ultrasound view at the long axis showing that the left sacrotuberous ligament (STL) is seen as a hyperechogenic structure that continues caudally with the tendon of the long head biceps femoris (LHBF). Also, the tendon of the semimembranosus muscle is located as a hyperechogenic structure under LHBFB. GlMy: Gluteus maximus muscle (**B**) Anatomical dissection showing that the fibers of the left STL cross between them with a lambda (λ)-shaped bifurcation. Then, the superficial and medial side of the STL (white arrow) goes on caudally with the LHBF. Moreover, it is possible to observe that the lateral and the deeper part of the sacrotuberous ligament inserts at the ischial tuberosity (black *), showing an x shape. The deeper layers of the ligament (black *) insert at the ischial tuberosity (IT).

**Figure 8:**
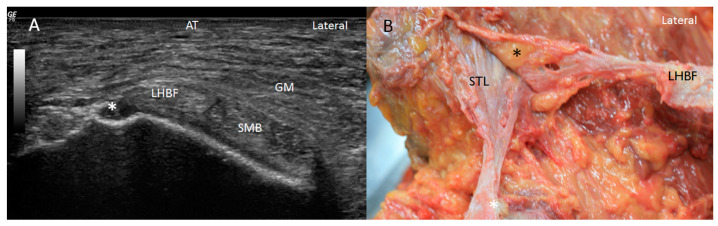
(**A**) Ultrasound view of the sacrotuberous ligament insertion at the vertex (white *) of the ischial tuberosity (IT) in the short axis tendon of the semimembranosus muscle (SMB) on the right side (gluteus maximus muscle). AT: Adipose tissue. (**B**) Anatomical dissection of the sacrotuberous ligament on the right side showing the possibility to separate different layers of the STL. The most superficial layer of the STL (black *) follows with the LHBF, and it is separated from the deep layer with adipose tissue (black *) and other layers of the STL inserted at the vertex of the IT (white *).

With respect to the STL, it was observed as a hyperechogenic structure under the gluteus maximus that continued with the LHBF in the short and long axes (Figure 7A). Moreover, it is possible to observe in the short axis a small fossa, just at the vertex of the ischial tuberosity, in association with the STL (Figure 8A).

**Figure 9:**
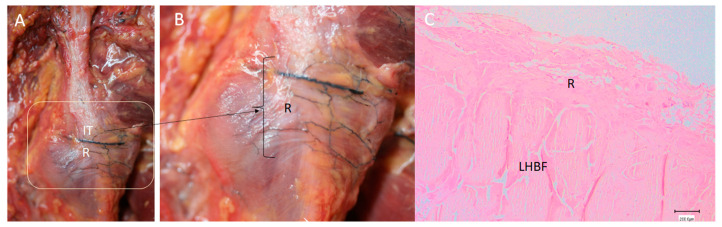
(**A**) Anatomical view of the retinaculum (R) at the ischial tuberosity (IT) on the right side. (**B**) It is possible to see the retinaculum increasing with the transversal fibers fixing the origin of the LHBF and SMB (in this case, it is possible to see a transversal black line corresponding to an injected vessel). (**C**) Histological study showing the retinaculum as dense connective tissue in a very close relation with the LHBF.

Dissection confirmed the findings of the ultrasound analysis. The SMB and LHBF originated at the lateral side of the ischial tuberosity, and a dense connective tissue, such as a retinaculum, kept the SMB and LHBF tendons fixed to the ischial tuberosity (Figure 1B and Figure 9A–C. When the retinaculum was cut, we observed that the injected dye, guided by ultrasound, was in the right tendon. However, the dye had spread superiorly in the specimens where the SMB did not have a well-defined structure (as seen in the ultrasound analysis) (Figure 2B). Although both tendons had a defined origin at the ischial tuberosity, they presented a tendinous continuity at the middle point of the ischial tuberosity. This contact was more evident at the point where the tendons crossed one another and stronger in the inferior part where the SMB tendon was towards the anterior side of the ST. It was necessary to cut the two tendons to isolate each one. The muscular fibers of the ST were not observed at this point, but a connection between the SMB tendon and the adductor magnus was observed in all the cases (Figure 6B,C).

**Figure 10:**
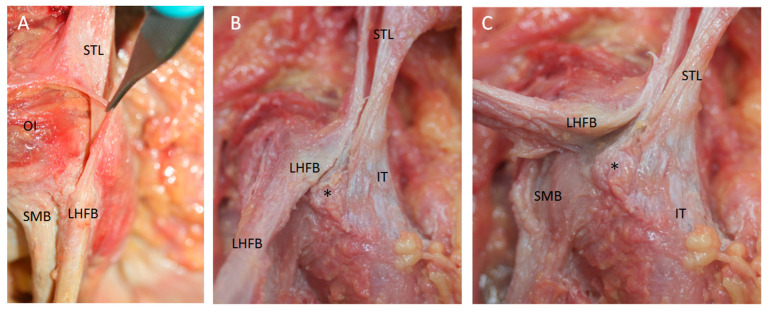
(**A**) The accurate dissection of the STL showing that the most superficial layer of the sacrotuberous ligament (STL) is continuous with the long tendon of biceps femoris (LHBF) superficially on the right side. OI: Obturator internus. (**B**) STL inserted at the LHBF as and also has a insertion as a cord in the vertex (black *) of the ischial tuberosity and at the medial side of the ischial tuberosity (IT). (**C**) After moving medially the LHBF it is possible to observe all the origin extension of the tendon of semimembranosus muscle (SMB).

After removing the LHBF tendon origin, proximal tendon attachment of the SMB at the lateral side of the ischial tuberosity could be accurately visualized (Figure 5B). It was longer and originated from the lateral side of the ischial tuberosity. It had a rounder shape at the more superior and lateral part, which distally became thinner, presenting an oval shape at the lateral side before becoming flattened medially to assume the shape of a teardrop (Figure 10 and Figure 12). It was covered posteriorly by the ST.

**Figure 11:**
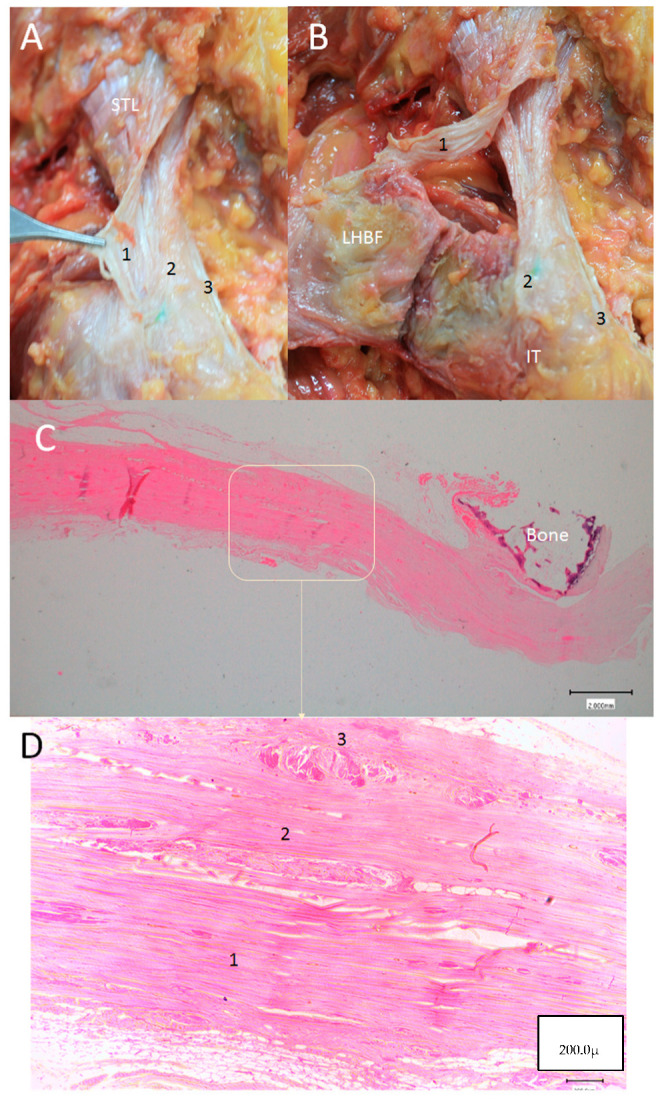
(**A**,**B**) The anatomical study showing different layers (1, 2, 3) of the sacrotuberous ligament (STL) that insert at different points on the right side. They have to be separated with a scalpel; however, it is very evident in the difference between layers 1 and 2. Layer 1 follows with the superficial tendinous fibers of the tendon of the long head biceps femoris (LHBF). Layer 2 inserts at the vertex of the ischial tuberosity and layer 3 at the medial side of the ischial tuberosity. (**C**) Microscopical study showing the dense connective tissue of the STL, seen transversally, and its relationship with the sacrum bone. (**D**) A higher vision of the STL allowing us to see the three layers separated, in this case, by dense, less-organize connective tissue.

The accurate dissection of the STL showed that it was formed of three layers separated sometimes by adipose tissue and with a lambda (λ)-shaped bifurcation (Figure 7B). The most superficial (or posterior) and more medial layer was continuous with the LHBF superficially (Figure 8B), while the medial layer was inserted as a cord in the vertex of the ischial tuberosity (Figure 10). The deepest and more lateral layer was inserted at the medial side of the ischial tuberosity and followed distally with the origin of the adductor magnus (Figure 11).

**Figure 12:**
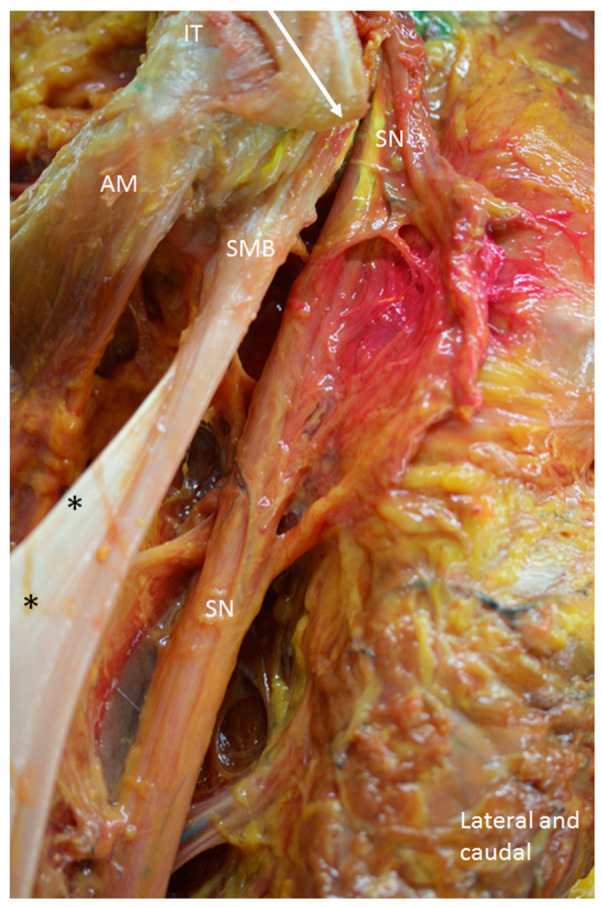
Anatomical view of the posterior and right side of the hip. The gluteus maximus muscle, the long head of the biceps femoris and the semitendinosus has been removed to observe the tendon of the semimembranosus muscle (SMB). It has a rounder shape at the more superior and lateral part (white arrow), which distally became thinner, and a thin membrane medially (black *) that originates at the lateral side of the ischial tuberosity (IT). AM: Adductor maximus muscles. The sciatic nerve (SN) at the lateral side has a close relationship with the SMB.

Anatomical cuts revealed the different anatomical structures. The tendinous origin of the SMB and LHBF had fibers in different directions. The SMB fibers had a vertical and transversal position, while the LHBF fibers were longitudinal. The inferior consecutive transverse anatomical cross-sections assumed the teardrop shape of the SMB muscle described in the dissection above. The distal cuts revealed that the diameter of the round and lateral portion decreased and became flattened distally, while the medial membranous portion of the SMB had a fascial connection with the adductor magnus and became wider and thinner (Figure 12).

Finally, an important relationship, seen via ultrasound, was confirmed via dissection and transversal cuts, namely, the presence of a thinner layer of dense connective tissue that came from the origin of the SMB and LHBF and ran transversally to reach the gluteus maximus muscle (Figure 4A). This layer made a specific tunnel to the passageway of the sciatic and posterior cutaneous nerves and isolated them from other structures (Figure 4B).

**Figure 13:**
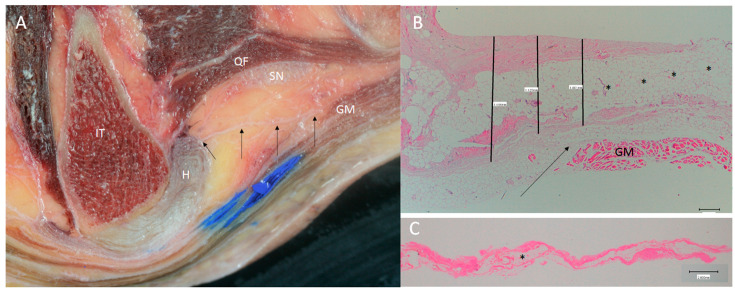
The expansion over the sciatic nerve (SN) (black arrows) was observed and measured: (**A**) anatomical view of the expansion going from the origin of the hamstrings (H) to the gluteus maximus muscle (GM) on the right side; (**B**) histological study allowing us to identify several thin and irregular layers of the dense connective tissue separated by adipose tissue (black *) (200.0 µ). (**C**) histological view of the expansion with similar morphology (2.000 mm).

The histological analysis revealed the presence of the three layers (Figure 11C,D) of the STL that were very close together but separated by adipose tissue, blood vessels or loose connective tissue. It was not possible to distinguish this ligament from the LHBF tendon, but it was possible to identify the different directions of the dense connective tissue fibers forming the tendons of the LHBF and SMB. The retinaculum (Figure 3B and Figure 9C) and expansion (Figure 5 and Figure 13) confirmed the dense connective tissue forming them, and their thicknesses were observed via ultrasound.

## 4. Discussion

The present study demonstrates that the proximal attachment of the hamstring muscles is more complex than that described in the classical anatomy textbooks. An ultrasound study can identify all the structures that are in association with them. Previous anatomical research focused on studying the morphology, morphometry and composition of these muscles. However, before the present study, the analysis of the proximal attachment of these muscles and the visualization of the connections between the hamstring, the ST, the adductor magnus and the sciatic nerve using ultrasound, macroscopic and microscopic analysis had not been performed. This information could help in understanding the causes of injury, explain certain pathologies associated with hamstring injuries or help in diagnostic examinations and even in therapeutic applications.

Several studies in this area [19] have described the common tendon of the ST and LHBF as an oval structure that measures 2.7 ± 0.5 cm from proximal to distal and 1.8 ± 0.2 cm from medial to lateral [12,19]. Other studies have also focused on morphology but have added the footprint of the proximal attachment of these muscles [11]. The length of the common tendon (LHBF and ST) is between 9.1 and 10 cm [11,19] (Feucht et al., 2015; Miller et al., 2007). The results are always very similar regardless of whether they were analyzed using anatomical samples or living volunteers [20]. More recently, the proximal/distal area ratio and the internal structure of the hamstrings have also been explored [21]. Due to the important and precise morphometric details of previous studies, the present investigation focused more on the relationships or connections of the proximal attachment of the hamstring muscles for possible clinical implications.

Some studies have observed a musculotendinous junction of the LHBF-ST and have described a common tendon between the LHBF and ST (showing a pennation angle of 9.2 ± 1.5 degrees, with muscle cells from both muscles interposing with the tendinous tissue) as an intrinsic risk factor for hamstring injury [10]. However, our dissections also revealed a close tendinous relationship between the superior origin of the LHBF and the STL and the SMB that must also be present in possible pathologies of this area.

The relationship of the LHBF with the STL has been described as an anatomical and histological continuity [8,9]. Our results further confirm this continuity through ultrasound, anatomical and histological findings. Moreover, some studies have described a continuity of the STL with the ST [9], even via MR imaging [22]. However, we believe that the dense connective tissue of the STL finishes with the origin of the LHBF, as described in other studies [8].

Unlike previous studies that have described the STL as consisting of two different layers without any muscular contributions [23], we observed that the STL can be formed of three layers, originating at the sacrum and presenting different attachments, as identified in the dissection and histological analysis. These different insertions could explain why some of the injuries of the hamstring muscles occur at the sacrum or even at the sacroiliac joint, which could be important for differential diagnoses. Moreover, one of the insertions is in association with the adductor magnus muscle origin, which could explain the important relationship between the pelvic position and lumbar biomechanics [24]. The present study would also suggest that the ischiocondylar portion of the adductor magnus muscle has a stabilizing function and a sizable tendon that can remain intact in proximal hamstring avulsions, as proposed by other studies [20,25]. This can also anatomically explain why there is an association between the adductor magnus muscle and hamstring pathology observed in clinical studies [26]. The connections of the attachment of the adductor magnus muscle and the SMB described in the present study would be a possible reason for the elevated incidence of hamstring injuries and also implies that injuries can occur at the adductor magnus muscle origin. These injuries account for 10% of all on-field injuries in team sports, with 13% of athletes sustaining a hamstring injury within 9 months, most commonly during competition [2]. Of all the hamstrings muscles, the one with the highest percentage of injuries is the LHBF [27]. This could be due to the LHBF presenting more fixation points. For example, in addition to being in continuation with the STL [8], the LHBF is fixed to the ischial tuberosity by a retinaculum [7], which was measured for the first time via ultrasound and histology in this study, and is also fixed, first with the SMB tendon and then with the ST tendon.

Injuries of the SMB muscle are the least common among the hamstring muscles [6,28,29] and are generally caused by hip elongation [30], which affects the cranial part of this muscle [6]. The present study describes the morphology of the SMB origin and would try to explain why it is less likely to become injured. Furthermore, its relationship with the adductor magnus could explain the injuries that occur with maximum hip extensions.

We did not analyze the expansions that the STL receive, but there is a difference in the attachment of the muscles of the superior part compared with that of the muscles of the inferior part that could explain certain pathologies. At the superior border of the ligament, some studies have described the convergence of the aponeurosis of the erector spinae muscles, the posterior wall of the para-spinal sheath (or posterior layer of the thoracolumbar fascia), and the gluteus maximus muscle before arrival at the STL [31]. At the inferior part, there are different points of anchoring on the ligament for the ST, the LHBF and adductor magnus. We postulate that this could be a stressful factor for the STL as it is composed of regular collagen that can hold maximal force in a limited number of planes, making it vulnerable to tensions or shear forces in different directions [32]. This could affect the LHBF given the connections between them.

Finally, some studies have used histology to observe the hamstring origin and the STL. Our findings are consistent with the results of other studies [9], revealing a distinct connection of the STL, LHBF and SMB on the ischia tuberosity, as well as an attachment of the STL to the LHBF, as described in some studies [8]. More studies are needed to define the level of collagen and elastin pathology, as well as the effects of this insertion in the STL on biomechanism.

The fascial structures covering the hamstrings and sciatic nerve were measured via ultrasound and histology for the first time in the present study; thus, we cannot compare these findings with those of other studies in the literature. The expansion covering the sciatic and posterior femoral cutaneous nerves is very thin, and its observations coincide with those of previous anatomical studies [7]. We would like to highlight that this fascial structure could be used for a sciatic nerve block, which has been described for other areas and other nerves [33,34,35,36], with lower risk of nerve damage. However, the fibrosis of these fascial expansions can be observed in hamstring syndrome and could cause nerve compression at this level [37]. Thus, regarding the clinical importance of this fascial expansion, the ultrasound study of this structure could be fundamental in the treatment of the neuropathies of these nerves.

The present study shares the limitations of other anatomical studies. First, it is a descriptive study that evaluated specimens with an advanced age. However, there is no evidence to suggest that muscle morphology and relationships change with age. Another limitation is that the functional role of these connections could not be analyzed with the specimens.

## 5. Conclusions

In conclusion, the proximal attachment of the hamstring muscles is more complex than classically described, and ultrasound is a valid tool with which to analyze anatomical structures. They are closely associated with the adductor magnus and the sacrotuberous ligament and important to clinical diagnosis.

## Figures and Tables

**Table 1:** Morphometry of the retinaculum (RT) and expansion over the sciatic nerve (ESN) in mm.

Parameters	Thickness RT (mm)	Thickness ESN (mm)
Average (SD)	0.57 (0.094)	0.20 (0.08)
95% CI	0.54–0.60	0.18–0.22
Median (IQR)	0.56 (0.12)	0.17 (0.11)
Shapiro–Wilk Test	0.941; *p* = 0.029	0.894; *p* = 0.001

**Table 2:** Distribution of the thickness of retinaculum (RT) and expansion over the sciatic nerve (ESN) according to the sex.

	RT (mm)		ESN (mm)	
Sex	Average (SD)	95%CI	Average (SD)	95%CI
Man	0.58 (0.098)	0.54 to 0.61	0.20 (0.08)	0.17 to 0.24
Woman	0.55 (0.086)	0.51 to 0.60	0.20 (0.07)	0.16 to 0.23
Mann–Whitney U Test	188.0; *p* = 0.411		233.0; *p* = 0.764	

**Table 3:** Distribution of the thickness of retinaculum (RT) and expansion over the sciatic nerve (ESN) according to the side.

	RT (mm)		ESN (mm)	
Side	Average (SD)	95%CI	Average (SD)	95%CI
Right	0.57 (0.11)	0.53–0.62	0.19 (0.06)	0.16–0.22
Left	0.56 (0.081)	0.53–0.60	0.21 (0.08)	0.18–0.25
Mann–Whitney U Test	232.0; *p* = 0.961		259.5; *p* = 0.469	

## Data Availability

The datasets used in this work can be accessed upon request.
